# LncRNA HSP90AA1-IT1 promotes gliomas by targeting miR-885-5p-CDK2 pathway

**DOI:** 10.18632/oncotarget.20777

**Published:** 2017-09-08

**Authors:** Taihong Gao, Guangyan Gu, Jingxia Tian, Rui Zhang, Xiangrong Zheng, Yanan Wang, Qi Pang, Qian Liu

**Affiliations:** ^1^ Department of Neurosurgery, Shandong Provincial Hospital Affiliated to Shandong University, Jinan 250021, Shandong, China; ^2^ Department of Histology and Embryology, Shandong University School of Medicine, Jinan 250012, Shandong, China; ^3^ Department of Gynecology and Obstetrics, Jinan Central Hospital Affiliated to Shandong University, Jinan 250013, Shandong, China

**Keywords:** lncRNA, HSP90AA1-IT1, miR-885-5p, CDK2, glioma

## Abstract

It is well established that ncRNAs are emerging as important regulators in various types of cancers, however, their functions and contributions in cancers remain insufficiently defined. In this study, we reported the expression levels of a long noncoding RNA (lncRNA), named HSP90AA1-IT1 (HSP90AA1 intronic transcript 1), appeared to correlate with the pathological grades of gliomas and high level of HSP90AA1-IT1 indicated poor prognosis. Downregulation of HSP90AA1-IT1 in the glioma cell lines significantly suppressed cell viability, proliferation, EMT, invasion and migration in addition to an increase in apoptosis and aberrant cell cycle progression. The tumorigenic capacity of these cells *in vivo* were also inhibited. We further demonstrated that the oncogenic effects of HSP90AA1-IT1 could be mediated by a direct binding to miR-885-5p. Sharing the same binding sites with CDK2, a key regulator in gliomagenesis, HSP90AA1-IT1 competitively bound to miR-885-5p, thereby prevented CDK2 from miR-885-5p mediated post-transcriptional repression. Taken together, it is concluded that HSP90AA1-IT1, performs its function via regulating the development of gliomas through miR-885-5p-CDK2 signaling axis, and this has added new perspective to its role in tumorigenesis, thus providing potential therapeutic targets for glioma treatment.

## INTRODUCTION

Gliomas are the most common and aggressive malignant brain tumors, which account for 30% of all central nervous system tumors and 80% of all primary malignant brain tumors [1, 2]. They are characterized with highly infiltrative growth, making them elusive targets for effective surgical management and leading to tumor recurrence [3]. Despite advances in neuro-oncology to optimize treatment options, the prognosis of patients with gliomas still remains poor [4]. It is therefore in an urgent need to identify novel strategies and targets for the early diagnostics and therapeutics of gliomas [5].

In recent years, the role of non-coding RNAs in tumors has attracted increasing attentions, among which long non-coding RNAs (lncRNAs) are a diverse and poorly conserved category of RNAs that constitute a large number of the human transcriptome [6-8]. They are longer than 200 nucleotides. Although poorly described, it is recognized that lncRNAs are capable of all sorts of tasks such as genomic imprinting, cell fate determination, RNA alternative splice and chromatin modification [9]. In addition, lncRNAs are involved in numerous brain functions and have been increasingly implicated in the pathobiology of gliomas [10]. Several lncRNAs that are associated with glioma histological subtypes have been identified through integrative analysis of their expression profiles [11-14]. Although this analysis predicted that lncRNAs could be potential drivers of glioma progression, systemic study on the functions and mechanisms of lncRNAs are still lacking.

Another kind of non-coding RNAs that play an important role in tumors are microRNAs, which are 22-24 nucleotides long. Through binding to the microRNA response elements (MREs) on the 3’UTR of targeted mRNA, microRNAs are able to control gene expression for degradation or translational repression [15]. It has been reported that aberrant microRNA levels are also involved in the development of gliomas, which participate in various cellular events such as proliferation, apoptosis, differentiation and cell transformation [16-18]. Furthermore, emerging evidences suggest that cross-regulations exist between microRNAs and lncRNAs. Like mRNA, lncRNAs harbor MREs as well. They have the potential to sequester and release microRNAs from specific mRNA targets, thereby modulating the expression and biological functions of microRNAs [19, 20].

In this study, we identified and functionally characterized an lncRNA, HSP90AA1-IT1 in the development of gliomas. The expression pattern of HSP90AA1-IT1 appeared to correlate with the malignancy of glioblastomas and high level of HSP90AA1-IT1 indicated poor prognosis. Furthermore, downregulation of HSP90AA1-IT1 in the glioma cell lines significantly suppressed the proliferative and invasive potential of these cells. We have identified miR-885-5p as the potential downstream mediator of the above-mentioned effects. Results showed that HSP90AA1-IT1 directly bound to miR-885-5p and thereby prevented CDK2, a key factor in gliomagenesis, from miR-885-5p-mediated inhibition. Taken together, we propose that HSP90AA1-IT1, a potential oncogenic lncRNA, performs its function in part via regulating miR-885-5p-CDK2 signaling axis, which would provide a new therapeutic perspective on targeting gliomas.

## RESULTS

### HSP90AA1-IT1 was significantly upregulated in the primary glioma samples

**Figure 1:**
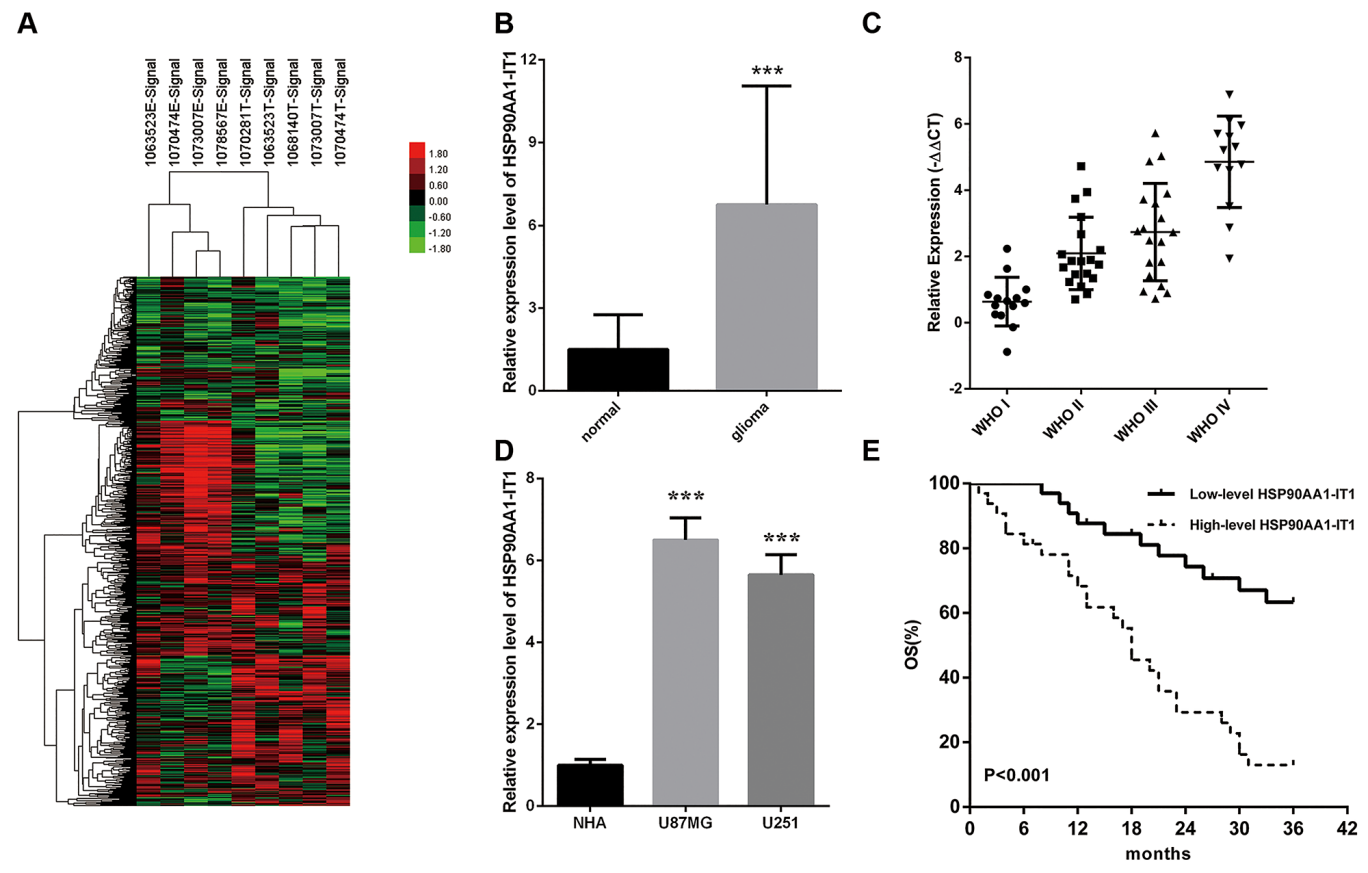
The expression profiles of HSP90AA1-IT1 in the glioma specimens and cell lines **(A)** The differential expression clusters of lncRNAs in the glioma specimens and paracarcinomic tissues were shown using high throughput technologies. The suffix E-Signal represented paracarcinomic tissues, and the suffix T-Signal represented tumor tissues. The red color indicated the upregulated lncRNAs and the green color indicated the opposite. The endogenous expression levels of HSP90AA1-IT1 in 133 clinical glioma specimens in total **(B)** or classified into four World Health Organization (WHO) grades **(C)** were determined by qRT-PCR. Data were presented as mean ± s.d. from three independent experiments. ***P < 0.001 vs. results of the normal brain tissues. **(D)** The expressions of HSP90AA1-IT1 in the two glioma cell lines were tested by qRT-PCR compared to normal astrocytes (NHA). Each bar represents mean ± s.d. from three independent experiments. ***P < 0.001 vs. NHA. **(E)** The overall survival of the 65 glioma patients were recorded based on low or high expression levels of HSP90AA1-IT1.

To identify novel lncRNAs involved in the development of gliomas, we carried out a lncRNA expression profile analysis using high throughput technologies. We identified the expression of lncRNAs in five glioma tissue samples and four paracarcinomic tissues by using Affymetrix GeneChip® Human Transcriptome Array 2.0. NONHSAT040076, which we named HSP90AA1-IT1 (HSP90AA1 intronic transcript 1) exhibited significant upregulation in the glioma tissues compared to the paracarcinomic tissues (Figure 1A). Subsequent qRT-PCR analysis in series of 133 clinical samples confirmed this result, as evidenced by a 5.96 -fold increase in the transcripts of HSP90AA1-IT1 in the tumor sections (P<0.001, Figure 1B). Furthermore the expression levels of HSP90AA1-IT1 were positively correlated with the pathological grades of these patients with gliomas (Figure 1C). In the glioma cell lines, HSP90AA1-IT1 was conceivably higher in comparative to normal human astrocytes (P<0.001, Figure 1D), further suggesting the potential role of HSP90AA1-IT1 in gliomagenesis.

### High level of HSP90AA1-IT1 was associated with poor prognosis of glioma patients

To estimate the epidemiological value of HSP90AA1-IT1 in the glioma patients, we divided 65 patients with gliomas, consisting of various grades, into two groups according to the relative expression levels of HSP90AA1-IT1. The cases which transcripts elevated at least 2-fold greater than normal brain tissues belonged to the high-level HSP90AA1-IT1 group. The rest of the patients belonged to the low-level HSP90AA1-IT1 group. Table 1 compared the demographic characteristics of the overall population. Among the 32 high-level group, there were 19 males and 13 females with an average age of 41.6±14.2, whereas in the 33 low-level group, 20 males and 13 females with an average age of 32.9±18.3 were included. No statistically significant differences were found regarding age, gender, and tumor sizes. In contrast, the expression levels of HSP90AA1-IT1 were significantly elevated in the gliomas with deteriorating differentiation states (r=0.584, P=0.000), evidently consistent with our previous results. Likewise, the amounts of HPS90AA1-IT1 and Ki-67 were positively correlated (r=0.353, P=0.004).

**Table 1:** Demographic characteristics of the overall glioma population

Factor	Total	HSP90AA1-IT1 expression		r	P-value
		Low	High		
**All case**	65	33	32		
**Age at surgery**					
**≥40**	32	14	18	0.138	0.272
**<40**	33	19	14		
**Sex**					
**Male**	39	20	19	-0.013	0.921
**Female**	26	13	13		
**WHO Grades**					
**I**	14	13	1	0.584	0.000
**II**	19	12	7		
**III**	19	7	12		
**IV**	13	1	12		
**Tumor Size**					
**≤ 3 cm**	26	15	11	0.102	0.418
**3-5 cm**	28	13	15		
**> 5 cm**	11	5	6		
**Ki 67**					
**≤ 10%**	28	20	8	0.353	0.004
**10%-20%**	23	9	14		
**> 20%**	14	4	10		
**Death**					
**No**	27	22	5	0.518	0.000
**Yes**	38	11	27		

During the three year follow-up period, 38 of the 65 glioma patients within the investigation (58.5%) have died, among which 27 (84.4 %) from the high-level group and 11 (33.3 %) from the low-level group (Table 1). The overall survival of the glioma patients with high-level of HSP90AA1-IT1 was obviously lower than that with low-level expression (Figure 1E). Cox’s proportional hazards regression model analysis indicated that HSP90AA1-IT1 (hazard ratio, HR=4.166, P=0.000) was a significant predictor for the prognosis of gliomas in the overall population of this study (Table 2).

**Table 2:** Univariate and multivariate analysis of glioma

Variables	Univariate analysis			Multivariate analysis		
	HR	95% CI	P-value	HR	95% CI	P-value
**Age at surgery**	1.407	0.738-2.685	0.300	-	-	-
**Sex**	1.259	0.634-2.497	0.510	-	-	-
**WHO Grades**	2.172	1.544-3.057	0.000	1.604	1.072-2.401	0.022
**Tumor Size**	1.046	0.869-1.259	0.632	-	-	-
**Ki 67**	2.469	1.604-3.800	0.000	2.186	1.358-3.518	0.001
**HSP90AA1-IT1**	4.166	2.046-8.479	0.000	2.471	1.123-5.434	0.024

### HSP90AA1-IT1 affected the viability of the glioma cells

To shed light on whether HSP90AA1-IT1 is indeed involved in gliomagenesis, we infected lentiviruses expressing small hairpin RNAs (shRNAs) of HSP90AA1-IT1 into the U87MG and U251 glioma cells. Lentiviral vectors with nonspecific shRNAs was taken as the negative control. qRT-PCR analysis indicated that a decrease of about 85% was achieved in the sh-HSP90AA1-IT1 infected cells compared to the scrambled-shRNA infected cells (Data not shown).

**Figure 2:**
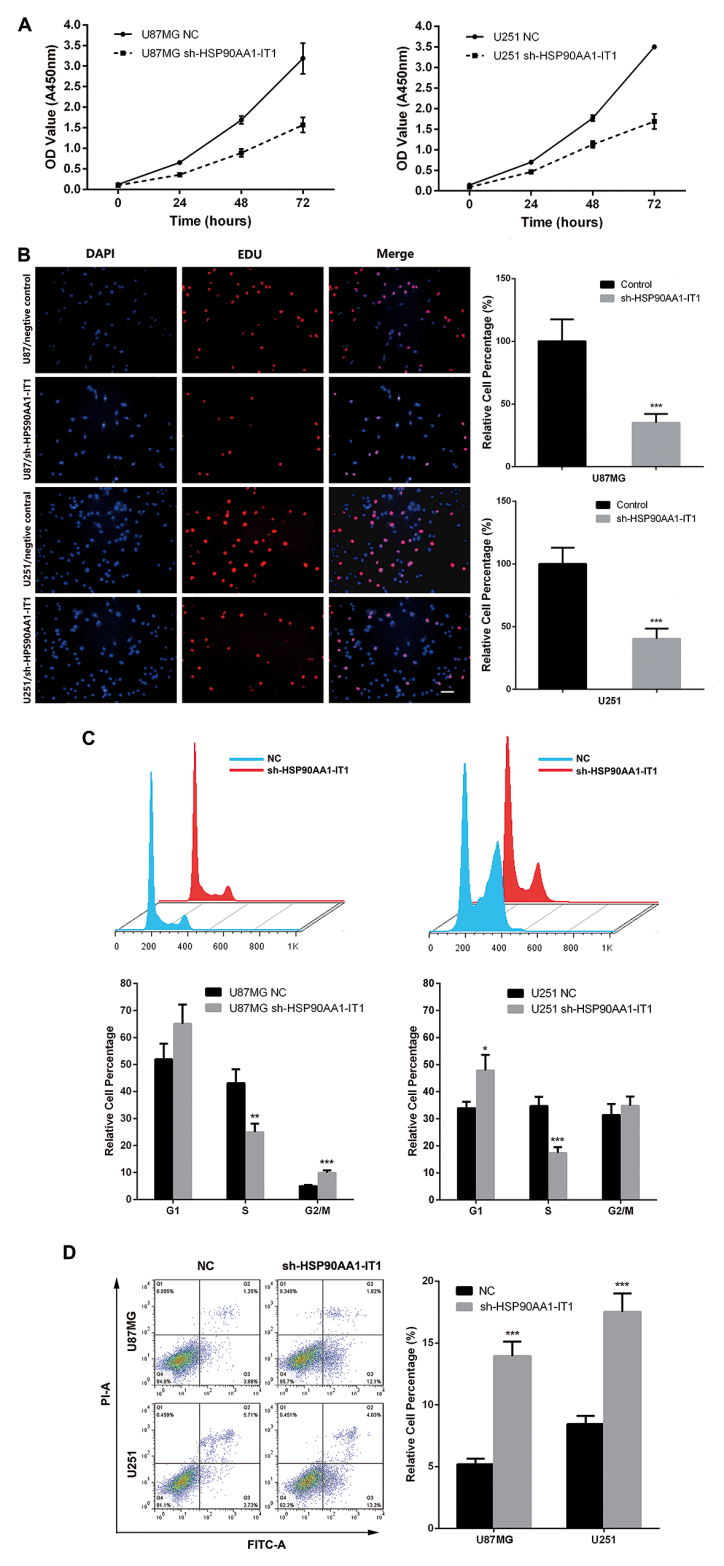
Effects of HSP90AA1-IT1 on the viability of the glioma cells The U87MG and U251 glioma cells were infected with lentiviruses expressing small hairpin RNAs of HSP90AA1-IT1 (sh-HSP90AA1-IT1). Lentiviral vector with nonspecific shRNAs was taken as the negative control (NC). **(A)** The growth curves of the infected glioma cells were shown using CCK8 assay. Data were presented as mean ± s.d. from three independent experiments. **(B)** Cell proliferations were determined by EDU staining assay. The results represent mean ± s.d. from three independent experiments. ***P < 0.001 vs. NC. **(C)** Cell populations in G1, S and G2/M phases were determined by flow cytometry. Each bar represents mean ± s.d. from three independent experiments. **P < 0.01 vs. NC, ***P < 0.001 vs. NC. **(D)** Apoptosis were tested by duel-staining with Annexin V and PI followed by flow cytometry assay. Data were presented as mean ± s.d. from three independent experiments. ***P < 0.001 vs. NC. Scale: 20μm.

A traditional CCK8 assay was performed to evaluate whether HSP90AA1-IT1 would affect viability of the glioma cells. As the result shown in Figure 2A, cell growth was markedly inhibited in both of the U87MG and U251 cells when HSP90AA1-IT1 was downregulated (Figure 2A). To further clarify whether reduction of cell viability was resulted from changes in cell proliferation or apoptosis, EDU cell proliferation assay was applied. As depicted in Figure 2B, number of EDU positive cells was significantly decreased when HSP90AA1-IT1 was knocked down in both cell lines (Figure 2B). What’s more, depletion of HSP90AA1-IT1 resulted in a marked reduction in the percentage of cells in S phase and increased that in G1 phase, suggesting an important role of HSP90AA1-IT1 in G1/S transition (Figure 2C). A subsequent duel-staining with Annexin V and PI followed by flow cytometry assay indicated that 16.4% of U87MG and 8.8% of U251 cells underwent apoptosis with downregulation of HSP90AA1-IT1; while only 8.9% and 4.1% of apoptotic cells were observed in the cells infected with the negative control (Figure 2D). Collectively, these data suggested that HSP90AA1-IT1 was indeed involved in the proliferative ability of gliomas by affecting both cell cycle progression and apoptosis.

### Knockdown of HSP90AA1-IT1 inhibited the migration, invasion and EMT of the glioma cells

**Figure 3:**
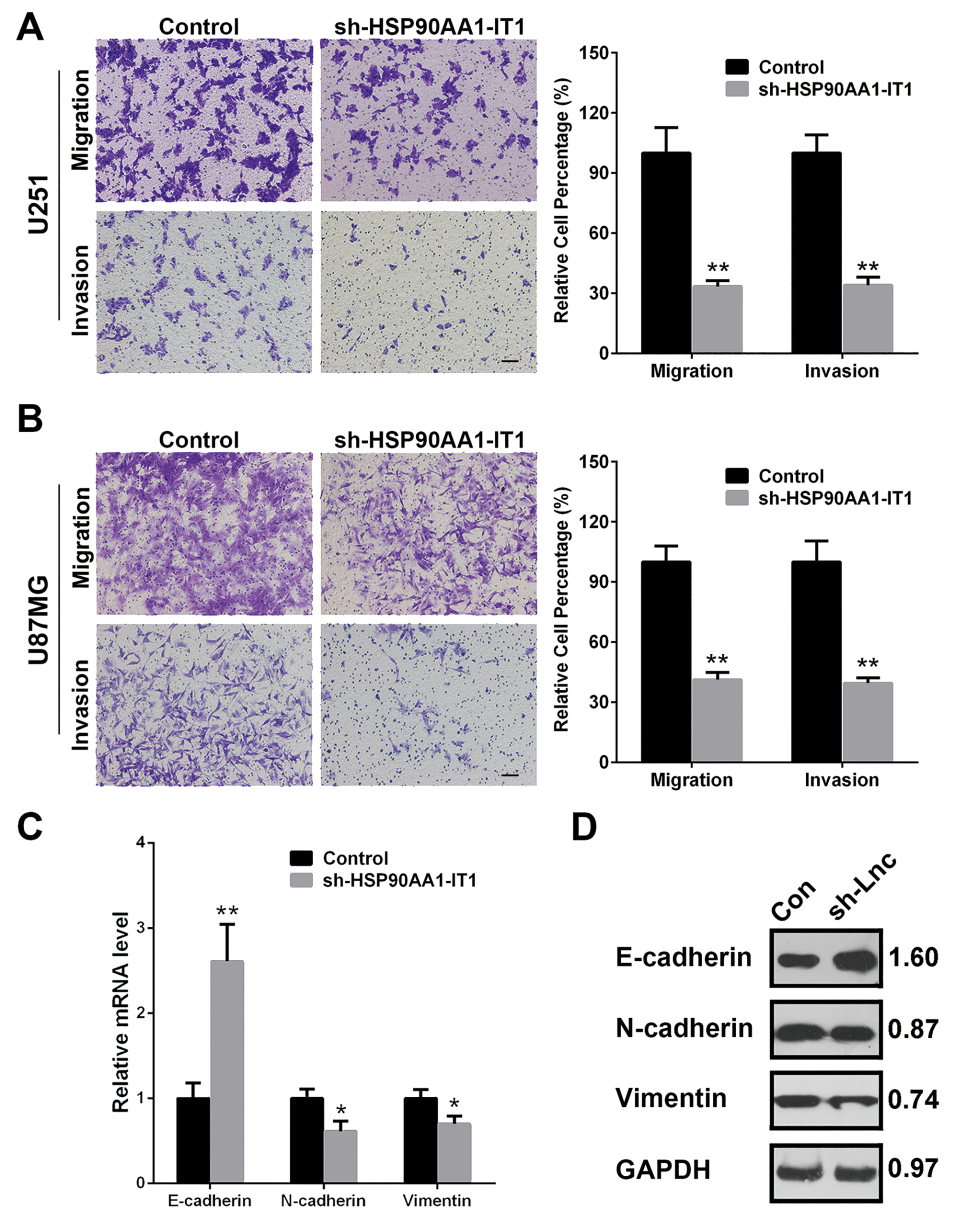
Effects of HSP90AA1-IT1 on the migration/invasion of the glioma cells The U87MG and U251 glioma cells were infected with lentiviruses expressing sh-HSP90AA1-IT1. Lentiviral vector with nonspecific shRNAs was taken as the NC. **(A, B)** Cell migration and invasion were determined by transwell assay. Each bar represents mean ± s.d. from three independent experiments. *P < 0.05 vs. NC, **P < 0.01 vs. NC. The mRNA **(C)** and protein **(D)** levels of the representative EMT markers including E-cadherin, N-cadherin and Vimentin were measured by qRT-PCR and Western Blotting analyses respectively. GAPDH was used as an internal control. The results represent mean ± s.d. from three independent experiments. *P< 0.05 vs. NC, **P< 0.01 vs. NC. Scale: 20μm.

As one of the most aggressive malignant brain tumors, gliomas are characterized with massive infiltrative growth without boundary from surrounding tissues. Therefore, to investigate whether HPS90AA1-IT1 contributes to the migration and invasion of glioma cells, transwell assay was applied in the transfected U87MG and U251 cell lines. The results showed that number of the migrating or invading cells with HSP90AA1-IT1 knockdown was significantly decreased compared to that of the scrambled cells, with average reduction rate of 65.98% for the U251 cells and 60.51% for the U87MG cells, respectively (Figure 3A, 3B). Given that depletion of HSP90AA1-IT1 suppressed the migratory and invasive abilities of the glioma cells, we investigated the effect of HSP90AA1-IT1 on the EMT, a critical event in tumor invasion. qRT-PCR was performed to test expression levels of several molecular markers of the EMT, among which the epithelial markers such as E-cadherin was higher in the HSP90AA1-IT1 depleted cells than that in the scrambled cells, whereas the mesenchymal markers such as N-cadherin and vimentin were decreased (Figure 3C). Analysis of Western Blotting subsequently confirmed the above results, evidently strengthening an important role of HSP90AA1-IT1 in the EMT as well as the infiltrative ability of gliomas (Figure 3D).

### Reciprocal repression existed between HSP90AA1-IT1 and miR-885-5p

**Figure 4:**
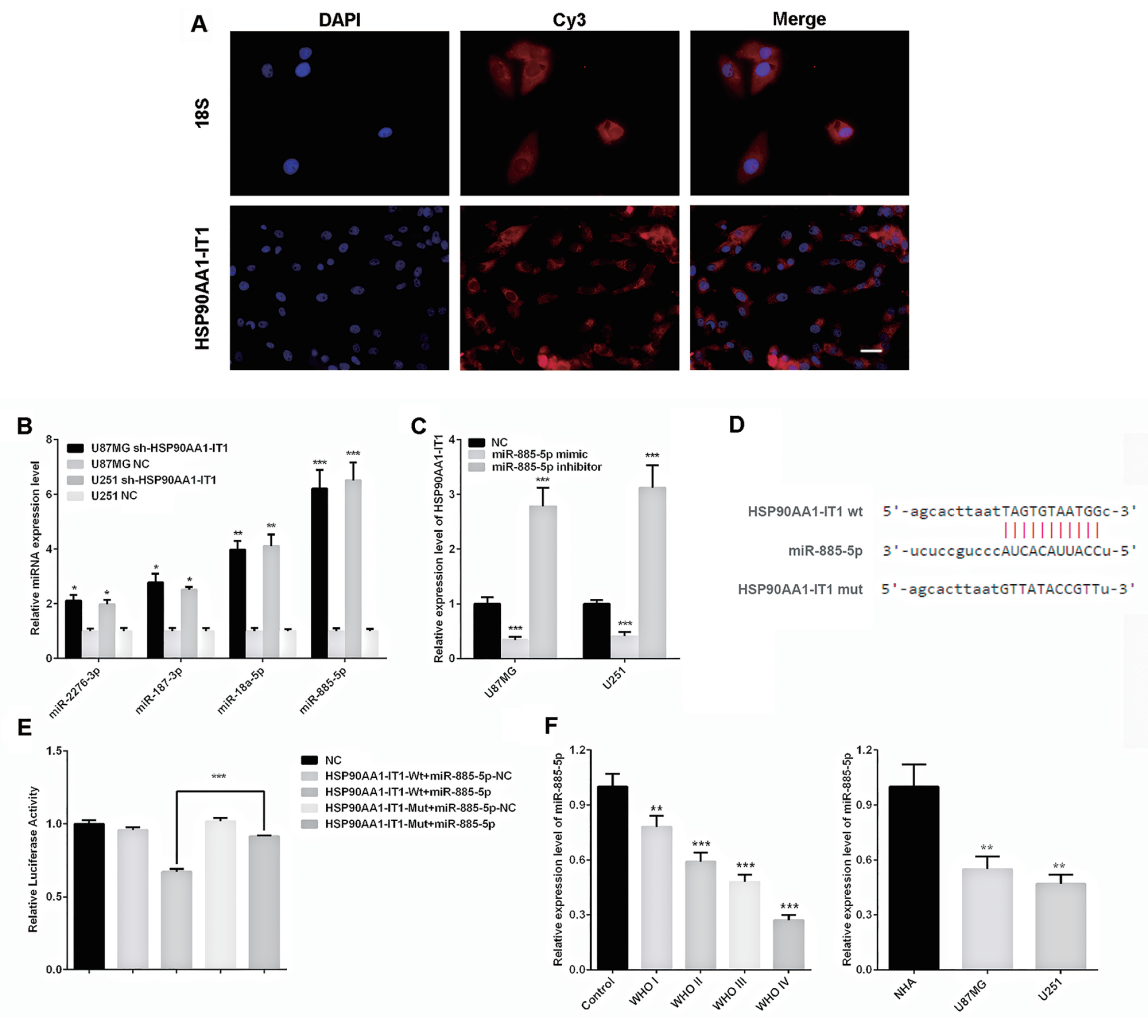
Reciprocal repressions between HSP90AA1-IT1 and miR-885-5p **(A)** Fluorescence in situ hybridization (FISH) assay showed that lncRNA HSP90AA1-IT1 mainly located in the cytoplasm of the glioma cells. Human 18S was used as an internal control. The lncRNA HSP90AA1-IT1 and h-18S were stained red by Cy3, and the nucleus were stained blue by DAPI. **(B)** The U87MG and U251 glioma cells were infected with lentiviruses expressing sh-HSP90AA1-IT1. Lentiviral vector with nonspecific shRNAs was taken as the NC. The expression levels of four potential miRNAs targeted on HSP90AA1-IT1 were determined by qRT-PCR. Data were presented as mean ± s.d. from three independent experiments. *P < 0.05 vs. NC, **P < 0.01 vs. NC, ***P < 0.001 vs. NC. **(C)** The U87MG and U251 glioma cells were transfected with miR-885-5p mimic or inhibitor. Non specific RNA oligonucleotides were taken as the NC. The expression levels of HSP90AA1-IT1 were tested by qRT-PCR. The results represent mean ± s.d. from three independent experiments. ***P < 0.001 vs. NC. **(D)** The potential binding sequence between HPS90AA1-IT1 and miR-885-5p was shown based on the target prediction analysis. **(E)** Relative luciferase activities were determined by dual-luciferase reporter assay. Data were presented as mean ± s.d. from three independent experiments. ***P < 0.001 vs. NC. **(F)** The endogenous expression levels of miR-885-5p in 133 clinical glioma specimens classified into four WHO grades and two glioma cell lines were determined by qRT-PCR. Each bar represents mean ± s.d. from three independent experiments. **P < 0.01 vs. results of the normal brain tissues (normal)/ astrocytes (NHA), ***P < 0.001 vs. normal/NHA. Scale: 20μm.

Considering the broad involvement of HSP90AA1-IT1 in the viability, proliferation, apoptosis, invasion and migration of glioma cells, we next sought to determine the precise mechanisms underlying these effects. In 2011, Pandolfi firstly proposed that competitive endogenous RNAs (ceRNAs) were widely existed in the cells such as mRNAs, transcribed pseudogenes or lncRNAs, which could regulate the expressions of each other by competitive binding to the same miRNAs on the miRNA response elements (MREs) [20]. As such, we firstly examined the distribution of HSP90AA1-IT1 by using fluorescence in situ hybridization (FISH). The results showed that lncRNA HSP90AA1-IT1 mainly located in the cytoplasm of the glioma cells (Figure 4A), evidently suggesting the potential role of HSP90AA1-IT1 as a ceRNA. Furthermore, making use of two target prediction databases Targetscan and miRanda, we identified four potential miRNAs targeted on HSP90AA1-IT1. We next examined the expression levels of these four miRNAs in the U87MG and U251 cell lines that were depleted of HSP90AA1-IT1, among which miR-885-5p was the most elevated (Figure 4B). On the other hand, when miR-885-5p mimics were applied to these cells, the amount of HSP90AA1-IT1 was significantly decreased. In line with the above results, miR-885-5p inhibitors remarkably promoted the expression of HSP90AA1-IT1 (Figure 4C), suggesting a reciprocally repressive relationship between HSP90AA1-IT1 and miR-885-5p.

Based on the target prediction analysis, the potential binding sequence of HPS90AA1-IT1 and miR-885-5p was shown in Figure 4D. To further confirm their regulatory functions, we made luciferase reporter constructs of wild-type and mutated form of HSP90AA1-IT. The results of dual-luciferase assay indicated that miR-885-5p lost its inhibitory effects when the potential binding sequence on HSP90AA1-IT1 was mutated (Figure 4E). A comparable result was observed in U251 glioma cells (data not shown), validating a direct interaction between HPS90AA1-IT1 and miR-885-5p by this putative binding site.

We then assessed miR-885-5p expressions in 133 glioma specimens and the two types of glioma cells. qRT-PCR analysis showed that miR-885-5p was significantly downregulated in the glioma tissues as compared to the normal brain tissue samples (P<0.01). Remarkably, the expression levels of miR-885-5p decreased progressively with deteriorating differentiation states and clinical stages, a trend exactly opposite with HPS90AA1-IT1. When we measured miR-885-5p levels in the U87MG and U251 cells, we found that the expressions of miR-885-5p were much lower in the glioma cell lines in comparative to the normal astrocytes (Figure 4F). Both results evidently supported that there was indeed inverse correlation between expressions of HPS90AA1-IT1 and miR-885-5p.

### HSP90AA1-IT1 compensated the negative effects of miR-885-5p on the proliferation and invasion of the glioma cells

**Figure 5:**
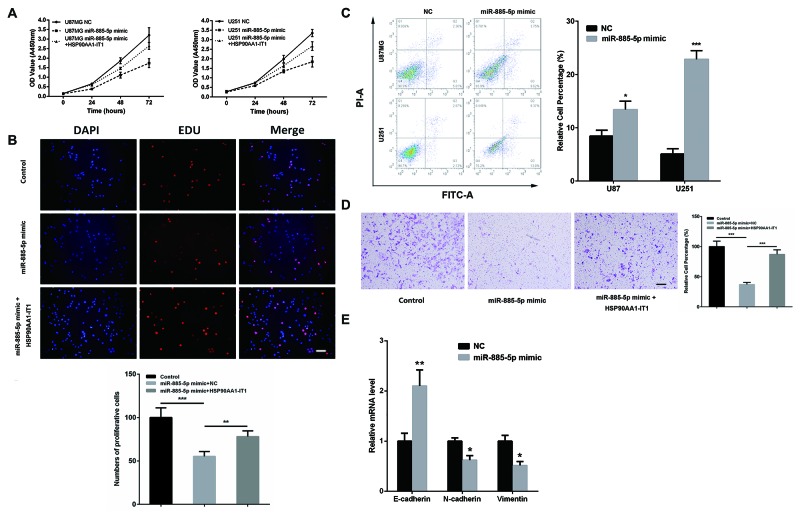
The inhibitory role of HSP90AA1-IT1 in the biological functions of miR-885-5p in the glioma cells **(A)** The U87MG and U251 glioma cells were transfected with miR-885-5p mimic alone or together with a lentiviral vector expressing HSP90AA1-IT1. The growth curves of the transfected cells were shown using CCK8 assay. Data were presented as mean ± s.d. from three independent experiments. The U87MG glioma cells were transfected with miR-885-5p mimic alone or together with a lentiviral vector expressing HSP90AA1-IT1. Cell proliferation **(B)** and invasion **(D)** were determined by EDU staining assay and transwell assay, respectively. The results represent mean ± s.d. from three independent experiments. **P < 0.01 vs. NC. **(C)** Both cell lines were transfected with miR-885-5p mimics. Apoptosis were tested by duel-staining with Annexin V and PI followed by flow cytometry assay. Data were presented as mean ± s.d. from three independent experiments. *P < 0.05 vs. NC, ***P < 0.001 vs. NC. **(E)** The U87MG glioma cells were transfected with miR-885-5p mimic. The mRNA levels of the representative EMT markers including E-cadherin, N-cadherin and Vimentin were measured by qRT-PCR. Each bar represents mean ± s.d. from three independent experiments. *P < 0.05 vs. NC. Scale: 20μm.

To step further to address whether miR-885-5p represented a functional link for the biological changes observed in the glioma cells depleted of HPS90AA1-IT1, we firstly investigated the specific functions of miR-885-5p in the glioma cells. When miR-885-5p mimic was transfected into the U87MG cells, cell viability (Figure 5A) and proliferation (Figure 5B) were significantly attenuated. Apoptosis, on the other hand, was greatly induced (Figure 5C). What’s more, upregulation of miR-885-5p resulted in the decreased invasion of these cells (Figure 5D) as well as aberrant expressions of the major molecular markers of the EMT (Figure 5E), evidently supporting the tumor suppressive effects of miR-885-5p in gliomagenesis. Most importantly, when HSP90AAA1-IT1 was overexpressed in the cells transfected with miR-885-5p mimic, the negative effects of miR-885-5p in glioma cell viability (Figure 5A), proliferation (Figure 5B) and invasion (Figure 5D) were significantly inversed. A similar phenomenon was manifested in the U251 cells (data not shown), further strengthening the inhibitory role of HSP90AA1-IT1 in the biological functions of miR-885-5p.

### HSP90AA1-IT1 regulated the expression of CDK2 through miR-885-5p

**Figure 6:**
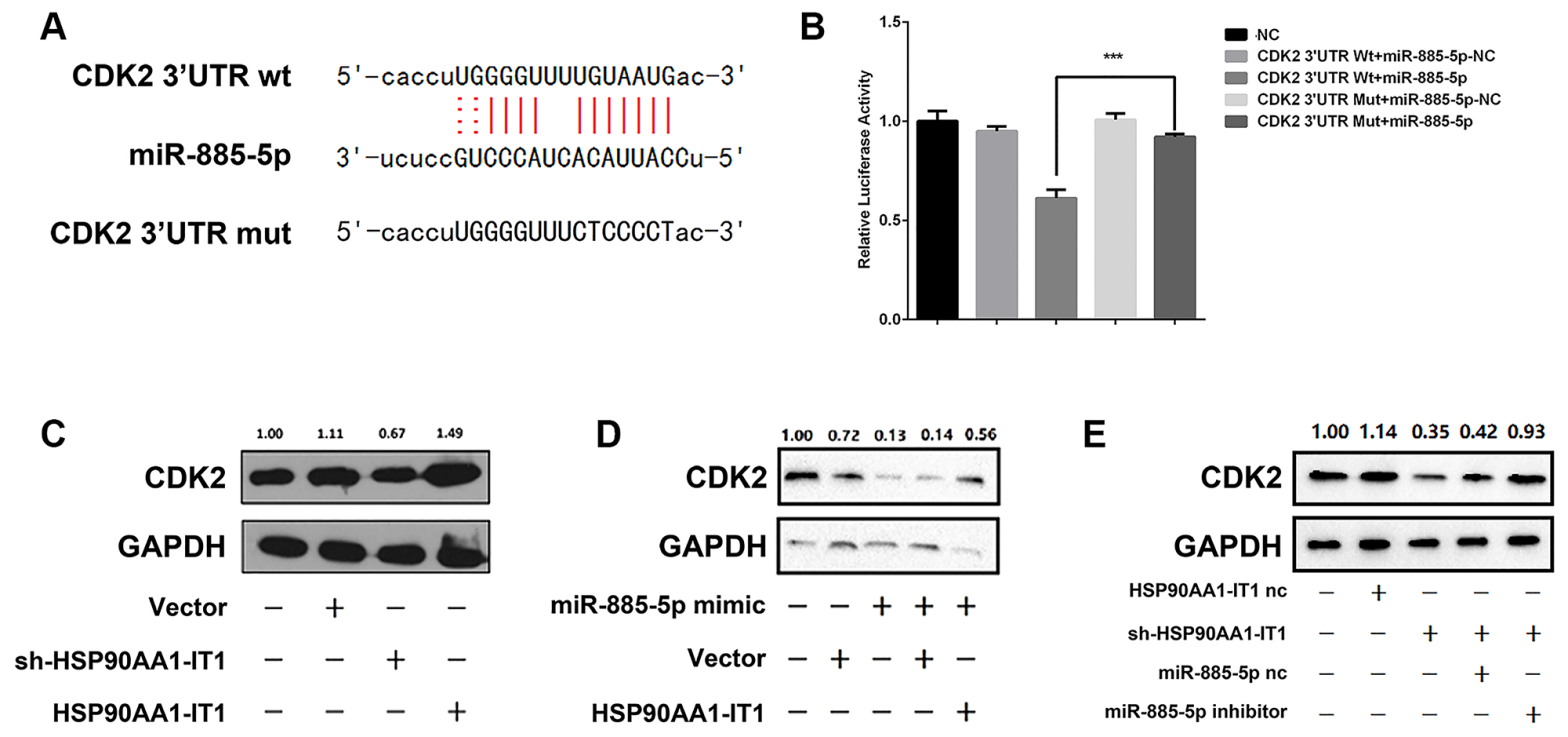
**(A)** The potential binding sequences of CDK2 and miR-885-5p as well as the mutated sequences were shown. **(B)** Relative luciferase activities were determined by dual-luciferase reporter assay. Data were presented as mean ± s.d. from three independent experiments. ***P < 0.001 vs. NC. **(C)** The U87MG cells were transfected with the lentiviral vectors expressing sh-HSP90AA1-IT1 or wild-type HSP90AA1-IT1. The protein levels of CDK2 were measured by Western Blotting analyses. GAPDH was used as an internal control. **(D)** The U87MG glioma cells were transfected with miR-885-5p mimic alone or together with a lentiviral vector expressing HSP90AA1-IT1. CDK2 expressions were determined by Western Blotting analyses. GAPDH was used as an internal control. **(E)** The U87MG cells were transfected with a lentiviral vector expressing sh-HSP90AA1-IT1 alone or together with miR-885-5p inhibitor. HSP90AA1-IT1-NC and miR-885-5p-NC are their respective controls. CDK2 expressions were determined by Western Blotting analyses. GAPDH was used as an internal control. Data are presented as the mean ± s.d. from three independent experiments.

In 2011, EAs’ group reported that miR-885-5p targeted cyclin-dependent kinase 2 (CDK2) and inhibited proliferation and survival of neuroblastoma [21]. CDK2, associated with its cyclins, are key factors in control of cell cycle progression. Aberrant expression of CDK2 has been associated with hyperproliferation, apoptosis, invasion and migration of cancer cells. By using the bioinformatic software RNAhybrid, starBase 2.0 and miRanda, we identified the sequence of CDK2 that miR-885-5p was potentially recruited to (Figure 6A). To elucidate whether CDK2 was indeed a functional target of miR-885-5p, dual-luciferase reporter assay was conducted. The results showed that significant decrease in luciferase activity of wild-type form of CDK2 was achieved when miR-885-5p mimic was applied to the U87MG cells. In contrast, the transcriptional activity of mutated form of CDK2 was barely suppressed in the same sets of transfected cells (Figure 6B). Altogether, these results suggested that miR-885-5p directly targeted CDK2 in the glioma cells.

Based on the above information derived from the bioinformatic softwares, we coincidently found that the MRE on HSP90AA1-IT1 bound by miR-885-5p shared almost the same sequence as the MRE on CDK2. As such, it was hypothesized that HSP90AA1-IT1 was able to regulate CDK2 through miR-885-5p as a ceRNA. To test this hypothesis, we first sought to determine whether the expression of CDK2 was indeed regulated by HSP90AA1-IT1. Western Blotting analysis showed that downregulation of endogenous HSP90AA1-IT1 in the U87MG cells significantly reduced the protein level of CDK2. On the other hand, an increase in CDK2 expression was coupled with upregulation of HSP90AA1-IT1 in the same cell lines (Figure 6C). Strikingly when HSP90AA1-IT1 was overexpressed into the glioma cells, the inhibitory effect of miR-885-5p on CDK2 was dramatically reversed, evidently supporting a ceRNA role of HSP90AA1-IT1 in the expression of CDK2 (Figure 6D). To further investigate whether miR-885-5p indeed worked as a mediator in the control of CDK2 by HSP90AA1-IT1, we knocked down the endogenous HSP90AA1-IT1 in the U87MG cells. The results showed that the decreased level of CDK2 resulted from depletion of HSP90AA1-IT1 was significantly attenuated in the presence of miR-885-5p inhibitors (Figure 6E). A comparable result was observed in the U251 glioma cells (data not shown). Taken together, all these data suggested that the oncogenic role of HSP90AA1-IT1 in gliomagenesis might be largely dependent on miR-885-5p-CDK2 axis.

### Knockdown of HSP90AA1-IT1 in combination with upregulation of miR-885-5p suppressed tumor growth *in vivo*

**Figure 7:**
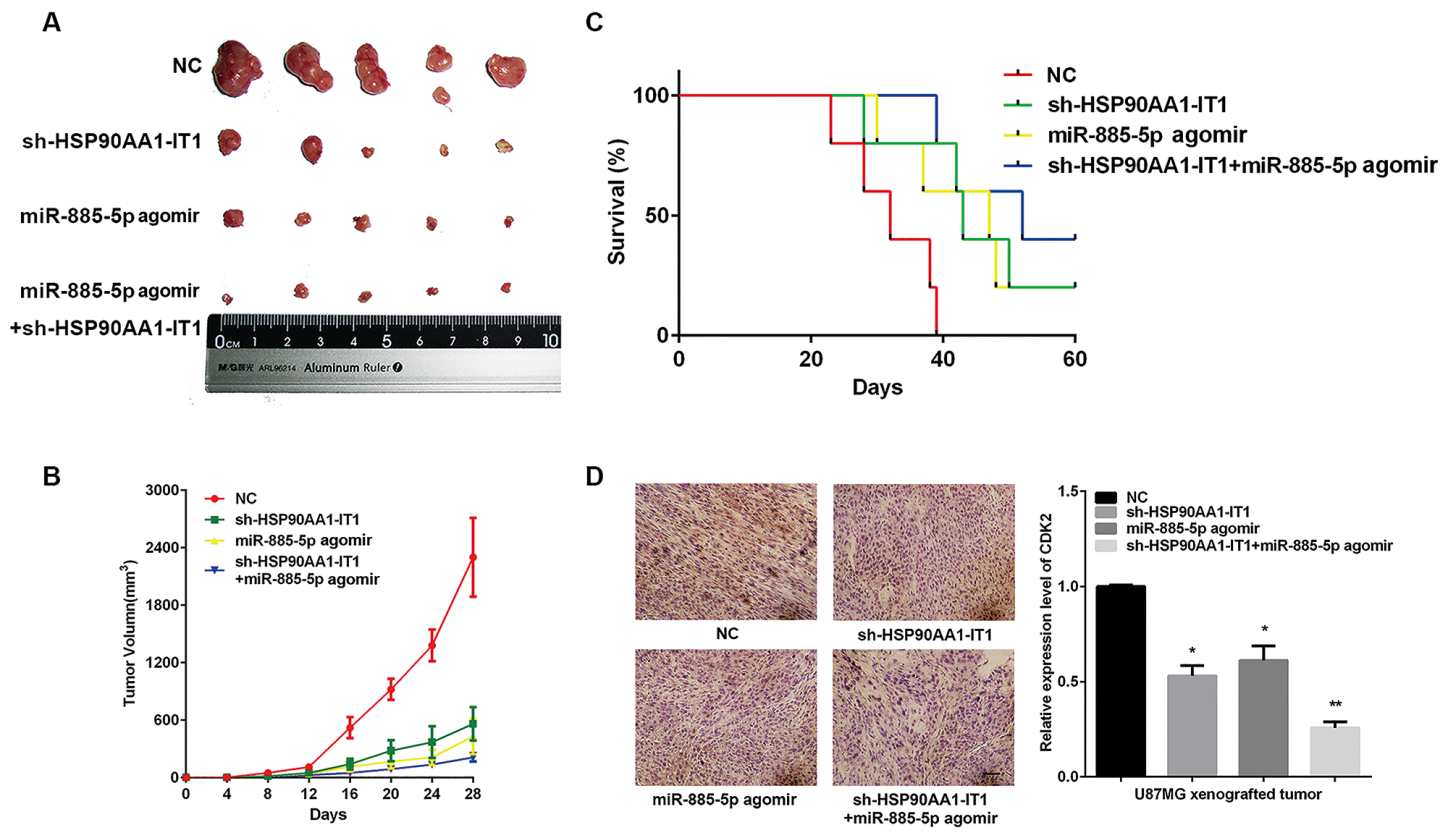
Effects of HSP90AA1-IT1 and miR-885-5p on tumorigenic capacities of the glioma cells *in vivo* The U87MG cells transduced with a lentiviral vector expressing sh-HSP90AA1-IT1, miR-885-5p agomir or both were subcutaneously implanted into the immunocompromised mice. N=20 in each group. **(A)** Representative xenograft tumors at 28 days after inoculation. **(B)** Growth curves of xenograft tumors were shown. Data were presented as mean ± s.d. from three independent experiments. **(C)** Curve graph indicated survival time of the xenograft mice. Log-rank test: Control group vs sh-HSP90AA1-IT1/miR-885-5p agomir group, P<0.005; Control group vs sh-HSP90AA1-IT1 group, P<0.05; Control group vs miR-885-5p agomir group, P<0.1. **(D)** Immunohistochemistry analysis was performed to determine the expressions of CDK2 in the xenograft tumors. Representative microscopic images were shown. Values in graphs represent mean of CDK2 densities ± s.d. The entire area of tumors similar in size from each group was photographed and included in the analysis. Scale: 20μm.

Considering the in *vitro* involvement of HSP90AA1-IT1 and miR-885-5p in glioma cell survival, proliferation, apoptosis and invasion/migration, we extended this study to determine the impact of HSP90AA1-IT1 and miR-885-5p on tumorigenic capabilities of glioma cells *in vivo*. When the U87MG cells transduced with the lentiviral vectors expressing shRNA targeting HSP90AA1-IT1 or non-targeting controls were subcutaneously implanted into the immunocompromised mice, we observed a significant decrease in tumor formation and an increase in the survival of tumor bearing mice when HSP90AA1-IT1 was targeted. Similar results were manifested in the xenografts when miR-885-5p agomir (similar to mimic, but could remain for 6-8 weeks *in vivo*) was transduced into these cells. Most strikingly, dual manipulation of the U87MG cells with shRNA targeting HSP90AA1-IT1/miR-885-5p agomir resulted in the smallest tumor volume and longest survival rate compared with the rest of the groups, supporting overlaid effects between downregulation of HSP90AA1-IT1 and upregulation of miR-885-5p (Figure 7A, 7B, 7C).

We next preformed immunohistochemistry on paraffin embedded mouse xenograft tumor samples with an antibody directed against CDK2. In contrast to higher CDK2 immunostaining in the gliomas derived from the cells transduced with scrambled control shRNA, the gliomas depleted of HSP90AA1-IT1 or upregulated with miR-885-5p displayed much lower and more variable levels of CDK2. Dual manipulation, in line with the above results, achieved lowest CDK2 expressions (Figure 7D), confirming the involvement of HSP90AA1-IT1/miR-885-5p/CDK2 signaling in the development of gliomas.

## DISCUSSION

Glioma is regarded as the most prevalent and malignant primary carcinoma in central nervous system. Hitherto, analyses of cancer development have focused mostly on protein-coding genes, but the function and contribution of non-coding RNAs remain insufficiently defined. HSP90AA1 intronic transcript 1 (HSP90AA1-IT1) is an lncRNA with 429 nucleotides, which locates at chromosome14q32.2 It is a transcript of HSP90AA1 retained an intron. Based on a large scale microarray expression profiling, we identified that HSP90AA1-IT1 was markedly upregulated in the glioma tissues compared with the corresponding peritumor tissues. Additionally its expression level was closely correlated with pathological grades of gliomas and negatively associated with prognosis of the glioma patients. Knockdown of HSP90AA1-IT1 in the glioma cell lines significantly suppressed cell viability, proliferation, migration, invasion and EMT in addition to an increase in apoptosis and aberrant cell cycle progression. The tumorigenic capacity of these cells were also inhibited. We further demonstrated that the oncogenic effects of HSP90AA1-IT1 could be mediated by a direct binding to miR-885-5p. Sharing the same binding sites with CDK2, a key factor in gliomagenesis, HSP90AA1-IT1 competitively bound to miR-885-5p, thereby prevented CDK2 from miR-885-5p mediated inhibition.

Noncoding RNAs play a vital role in the life courses of individuals. Their functions extend to all sorts of biological processes, such as embryonic development, cell proliferation, differentiation, apoptosis, gene imprinting, stress responses, and carcinogenesis. Recently, an increasing number of studies have been made on the mechanisms of lncRNAs in the initiation and development of gliomas. For instance, the lncRNA HOTAIR promotes glioblastoma cell cycle progression in an EZH2 dependent manner [22]. Introduction of the HOTAIR 5’ domain in human glioma-derived astrocytoma induced the expression of β-cantenin, resulting in elevated invasion/migration. H19, on the other hand, regulates glioma angiogenesis and the biological behavior of glioma-associated endothelial cells by inhibiting miR-29a [23]. Not only that, more and more lncRNAs have been identified in the progression of gliomas such as CASC2, MEG3, TUG1, CRNDE et al [11, 12, 14, 24]. However, the in-depth and detailed regulatory mechanisms are far from clarified. A better understanding of the interactions between various noncoding RNAs may shed light on new therapeutic strategies for gliomas.

In 2011, Pandolfi firstly proposed that competitive endogenous RNAs (ceRNAs) were widely existed in the cells, which sequestrate and release miRNAs from binding sites, known as miRNA response element (MRE). These MRE-containing platforms (transcribed pseudogenes, lncRNAs or circular RNAs) known as sponges relieve corresponding mRNA targets from repression or indirectly induce target mRNA repression by release of miRNAs from this reservoir [20].

Making use of two target prediction databases miRanda and Targetscan, we identified that miR-885-5p had a potential binding site on HSP90AA1-IT1. MiR-885-5p was located at 3p25.3 and has been characterized as a biomarker in several tumors. For instance, the level of miR-885-5p were significantly higher in pancreatic, colorectal and oncocytic thyroid cancer patients [25-27]. In contrast, enforced miR-885-5p expression suppressed cell survival in neuroblastoma and inhibited metastasis in hepatocellular carcinomas [21, 28]. Our results indicated that there was reciprocal repression between the expression levels of HSP90AA1-IT1 and miR-885-5p. MiR-885-5p mimics remarkably suppressed viability, proliferation, migration and tumorigenic capacity of glioma cells in addition to an increase in apoptosis, which could be attenuated by concomitant upregulation of HSP90AA1-IT1 These results evidently suggested that miR-885-5p could be involved in HSP90AA1-IT1-mediated gliomagenesis with a potential role as a tumor suppressor.

We also demonstrated that knockdown of HSP90AA1-IT1 significantly decreased the expression of CDK2. CDK2, associated with its cyclins, are key factors in control of the G1/S phase transition. Aberrant expression of CDK2 would cause abnormal regulation of cell-cycle, which is directly associated with hyperproliferation of cancer cells. What’s more, targeting CDK2 has been reported to inhibit invasion, migration together with outgrowth in various cancers. Based on miRanda, RNAhybrid and starBase2.0, we identified that HSP90AA1-IT1 and CDK2 shared the same potential binding site on miR-885-5p. Furthermore, the positive effect of HSP90AA1-IT1 on CDK2 expression levels could be reversed by miR-885-5p inhibitors, suggesting that HSP90AA1-IT1 might regulate CDK2 as a ceRNA and CDK2 could play an important role in HSP90AA1-IT1-mediated gliomagenesis through miR-885-5p.

In summary, we have for the first time provided unequivocal evidence demonstrating that HSP90AA1-IT1, a potential oncogenic lncRNA, performs its function in part via regulating gliomagenesis through miR-885-5p-CDK2 axis. The novel findings would provide a new perspective for glioma diagnosis and therapeutic intervention.

## MATERIALS AND METHODS

### Clinical specimens and cell lines

The 113 cases of clinical specimens were collected from tumorectomy of brain glioma in the Department of Neurosurgery, Shandong Provincial Hospital Affiliated to Shandong University, Jinan. The adjacent brain tissue was defined as 1cm away from the lesions. The criteria for the inclusion/exclusion of patient are: (1) age 0-70; (2) only received surgery, without preoperative chemotherapy or radiation therapy; (3) without any other type of cancer, autoimmune disease, infectious diseases, etc. All specimens were obtained under sterile conditions during surgery, snap frozen in liquid nitrogen, and stored at 80°C.

Primary normal human astrocytes (NHA) were purchased from the Sciencell Research Laboratories (Carlsbad, CA) and cultured under the conditions as instructed by the manufacturer. U87MG and U251 cells were obtained from Shanghai Institutes for Biological Sciences Cell Resource Center (Shanghai, China), and cultured in DMEM medium with high glucose and sodium pyruvate, supplied with 12% fetal bovine serum, 100 units/mL penicillin and 100μg/mL streptomycin.

### Reagents, antibodies and lentivirus infection

High-glucose Dulbecco’s modified Eagle medium (DMEM) and fetal bovine serum (FBS) were purchased from Gibco (Life Technologies, Carlsbad, CA, USA). Penicillin–streptomycin were purchased from Invitrogen (Life Technologies).

The HSP90AA1-IT1 cDNA was cloned into the Lenti-OE vector (Genechem, Shanghai, China) to generate HSP90AA1-IT1-overexpressing lentiviral. The shRNAs were cloned into GV428 vector carried by Lenti-OE (Genechem, Shanghai, China), and the lncRNA knockdown targeted sequences was as follows: HSP90AA1-IT1: 5’-GGAACAAGCTAGAGCACTT-3. The miR-885-5p mimic, inhibitor and agomir were obtained from Ribobio (Guangzhou, China). All constructs were verified by sequencing. U98MG and U251 cells were infected by the lentivirus, and incubating for 24 hours.

The Fluorescent In Situ Hybridization Kit and FISH Probe was obtained from Ribobio (Guangzhou, China).

Rabbit polyclonal anti- N-Cadherin (ab18203) antibody, mouse monoclonal anti-E-Cadherin (ab1416) antibody, mouse monoclonal anti-Vimentin (ab8978) antibody, Rabbit monoclonal anti-CDK2 (ab32147) antibody and mouse monoclonal anti-GAPDH (ab8245) antibody were obtained from Abcam (Cambridge, MA, USA).

### Quantitative reverse transcription PCR (qRT-PCR)

Total RNA was extracted with Trizol reagent (Gibco, Birmingham, MI, USA). The cDNA was synthesized from total RNA using PrimeScript RT reagent Kit with gDNA Eraser (Takara, Japan). Real-time PCR was performed using an ABI 7300 Fast Real-time PCR System (Applied Biosystems, Carlsbad, California, USA) with a SYBR Premix Ex Taq kit (Applied TaKaRa, Japan). The primer sequences were as follows: for HSP90AA1-IT1, 5’-GTGTTAGGCATGGCGTTGAG-3’ (forward) and 5’-TCAGTTTGGTCTTCTTTCAGGTGT-3’ (reverse); for CDK2, 5’-AACTGGCCCTTCTTGGA-3’ (forward) and 5’-TCGTCATCTGGCTCCC-3’ (reverse); for E-cadherin, 5’-GTACTTGTAATGACACATCTC-3’ (forward) and 5’-TGCCAGTTTCTGCATCTTGC-3’ (reverse); for N-cadherin, 5’-CTCCTATGAGTGGAACAGGAACG-3’ (forward) and 5’-TTGGATCAATGTCATAATCAAGTGCTGTA-3’ (reverse); for Vimentin, 5’-AGATCGATGTGGACGTTTCC-3’ (forward) and 5’-CACCTGTCTCCGGTATTCGT-3’ (reverse); for GAPDH, 5’-GCACCGTCAAGGCTGAGAAC-3’ (forward) and 5’-TGGTGAAGACGCCAGTGGA-3’ (reverse).

### Western bloting

Total proteins were extracted using lysis buffer containing 10 mmol/L Tris HCl (pH 7.4), 1% Triton X-100 and protease/phosphates inhibitors (Roche Diagnostics, Indianapolis, IN, USA), separated by 10% SDS-PAGE gel electrophoresis, transferred to PVDF membranes and probed with primary antibodies. The membranes were subsequently probed with horseradish peroxidase-conjugated secondary antibodies followed by development using an enhanced chemiluminescence detection system (Pierce, Rockford, IL, USA). Anti-GAPDH antibody was used to monitor the loading amount.

### Cell proliferation

Glioma cells were planted in 96-well plates at the density of 2500 cells per well for 24 hours. Cell viability rate was determined using the Cell Counting Kit-8 (Dojindo, Japan). Optical density value was measured at 450 nm. Each group set five replicated wells and CCK-8 assay was performed in three independently repeated experiments. EDU immunocytochemistry staining was performed by Cell-Light™ EdU Apollo *In Vitro* Imaging Kit (Ribobio, Guangzhou, China)

### Cell invasion assay

Cell transfected with HSP90AA1-IT1 siRNAs were cultured at about 80% confluence. Cells were starved in basal medium without fetal bovine serum for 16h. Matrigel cell invasion assay was carried out using the BD BioCoat Tumor Invasion System (BD Biosciences #354165) as recommended by the manufacturer. 5×10^4^ starved glioma cells were seeded into the apical chambers, followed by adding a chemoattractant (basal medium plus 10% FBS) to the basal chambers. After 24h incubation, cells in the upper chambers were carefully removed with a cotton swab and the cells that had traversed the membrane were fixed in methanol and stained with leucocrystal violet. The number of invasive cells was determined by counting the leucocrystal violet stained cells. For quantification, cells were counted under a microscope in five fields (up, down, median, left, right. ×200).

### Cell migration assay

Cell migration was determined by using a modified two chamber migration assay. For migration assay, 5×104 starved glioma cells were seeded in serum-free medium in the upper chamber. After 12 h incubation at 37°C, cells in the upper chamber were carefully removed with a cotton swab and the cells that had traversed the membrane were fixed in methanol and stained with leucocrystal violet. Migration cells were counted under a microscope in five fields (up, down, median, left, right. ×200).

### Flow cytometry

Annexin V-FITC apoptosis detection kit (BD Biosciences; San Jose, CA, USA) was used to analyze cell apoptosis. According to protocols, cells were collected after the dissociation with EDTA-free trypsin, and then washed with cold phosphate-buffered saline (PBS). Then, cells were stained in the binding buffer with Annexin V-FITC and PI for an incubation of 15min in the darkness. Cell cycle was determined by Cell cycle detection kit (BD Biosciences; San Jose, CA, USA). After dissociation and alcohol fixation, cells were stained by PI buffer. Flow cytometry analysis was performed immediately on the BD FACS Calibur (BD Biosciences).

### *In vivo* experiments

All experimental animal procedures were conducted strictly in accordance with the Guide for the Care and Use of Laboratory Animals, and approved by the Animal Care and Use Committee of the Shandong provincial hospital affiliated to Shandong University. The male BALB/c nude mice were obtained from Cancer Institute of the Chinese Academy of Medical Science, which were randomized divide into four groups in a blinded manner, each group including five 4-weeks-old nude mice.

For subcutaneous xenograft study, 5×10^5^cells were subcutaneously injected in the right flanks of nude mice. For survival analysis in orthotopic xenograft, the mice were anesthetized, and then 1×10^6^glioblastoma cells were inoculated stereotactically into the right striatum using a Hamilton syringe (Reno, NV, USA).

For histopathologic analysis, the subcutaneous xenografts were made into paraffin sections followed by immunohistochemistry. Subcutaneous xenografts were dehydration, embedding and slicing up at 8-μm thickness. Slides were incubated overnight at 4 °C with primary antibodies (anti-CDK2 diluted at 1:100).

### Luciferase constructs and transfection

A dual-luciferase reporter vector was used to generate the luciferase constructs. The putative binding sites and its homologous mutation sites in the 3’-UTR region of CDK2 mRNA and HSP90AA1-IT1 were amplified and cloned into pmiRGLO luciferase reporter plasmid. And then, cells were plated in 24-well plates, and transfected with 0.1μg of either pmiRGLO empty, pmiRGLO-HSP90AA1-IT1-wt-site, pmiRGLO-HSP90AA1-IT1-mut-site, pmiRGLO-CDK2-wt-site or pmiRGLO-CDK2-mut-site using Effectene (Qiagen), or miRNA mimics. Firefly luciferase activity and renilla luciferase activity was detected using a dual-luciferase assay system (Promega, Madison, WI, USA) over a period of 24 h. Firefly luciferase activity was normalized to Renilla luciferase activity as an internal transfection control. Transfections were performed in there independent experiments, and assayed in duplicate. Data were presented as the mean value ± standard deviations (SD) for triplicate experiments.

### Statistical analysis

Quantitative data are expressed as mean ± standard deviation (SD). GraphPad Prism (GraphPad Software, San Diego, CA) was used for data analysis. The Student t test or one-way analysis of variance (ANOVA) was used to assess significant differences between groups. The Chi-square test was used to analyze the relationship between categorical variables. P< 0.05 was considered statistically significant.
